# An Evaluation of Interactive mHealth Applications for Adults Living with Cancer

**DOI:** 10.3390/curroncol30080518

**Published:** 2023-07-25

**Authors:** Sydney Wasserman, Lydia Ould Brahim, Ameer Attiya, Eric Belzile, Sylvie D. Lambert

**Affiliations:** 1 St. Mary’s Research Centre, Montreal, QC H3T 1M5, Canada; sydney.wasserman@mail.mcgill.ca (S.W.); lydia.ouldbrahim@mcail.mcgil.ca (L.O.B.); eric.belzile@ssss.gouv.qc.ca (E.B.); 2 Ingram School of Nursing, Faculty of Medicine and Health Science, McGill University, Montréal, QC H3A 0G4, Canada; 3 School of Rehabilitation, Faculty of Medicine, Université de Montréal, Montréal, QC H3C 3J7, Canada; ameer.attiya@umontreal.ca

**Keywords:** mHealth, cancer, mobile applications

## Abstract

This study evaluated the quality and usefulness of interactive mobile health (mHealth) applications (apps) for adults with cancer. The PRISMA guidelines were followed to add rigor to the search, as well as to the data collection and analysis. The apps available in the most used app stores (Google Play and Apple) with interactive tailored features were identified. To supplement this, a Google web search was also conducted. The apps were evaluated for their quality using the validated Mobile App Rating Scale (MARS) and for their usefulness using a checklist of end users‘ desired features derived from the literature. The searches returned 3046 apps and 17 were retained for evaluation. The average quality score of the apps across the sample was 3.62/5 (SD 0.26, range: 3.14–4.06), with Outcomes4me scoring the highest. On average, the apps scored 50% (SD 2.5, range: 31–88%) on the usefulness checklist, with Cancer.net scoring the highest. The lowest-scoring categories were communications features on the usefulness checklist and “information” on the MARS, indicating areas for future work. The findings identified the apps of an acceptable quality and usefulness that could be recommended to those with cancer.

## 1. Introduction

A rapidly accelerating avenue to better meet the needs of the growing population of people with cancer is using mobile and wireless devices (primarily smartphones), known as mHealth [1,2,3]. Since their induction in 2007, the number of smartphone users has increased dramatically, exceeding 3.5 billion in 2020, with future use expected to grow [4]. There has been a similar surge in the development and download of mobile applications (apps), including mHealth apps [5]. Further fueled by the COVID-19 pandemic, it is estimated that, in 2021, over 300,000 mHealth apps were available for download on commercial platforms [1,6]. Surveys have indicated that approximately half of mobile phone users have downloaded a health-related app, with 30–65% using them on a daily basis [7,8].

Apps designed for those with cancer are no exception, with an ever-increasing number becoming available for download [9]. There has also been a parallel rise in the research on cancer-related apps [3,5,10]. With growing access to mobile devices, such apps offer many potential benefits to patients, including convenience, immediate access to health information [11], increasing awareness of their own health, and features that may support connecting with other patients or healthcare professionals [12,13,14]. Due to such advantages, mHealth apps have generally been found to be acceptable to patients [13,15].

Many mHealth cancer apps include interactive features, such as tracking medications, symptoms, and side effects [16]. Often, these interactive features allow patients to input information that is then used to tailor the app’s content. Such levels of interactivity and customization are not only preferred by patients [17], but may also increase engagement and prevent abandonment of use, a necessary condition of effectiveness [2,18,19,20,21,22]. Further, receiving tailored messages, in comparison to generic ones, can be more effective in impacting health behavior changes (e.g., exercise and smoking cessation) [23]. These interactive features may improve the communication with healthcare teams and support patients in playing an active role in their healthcare and self-managing their illness [14,24]. Despite their benefits, there remain numerous concerns related to the use of mHealth apps. For example, there is limited evidence to support their effectiveness [25], many do not align with the current clinical practice guidelines [5,25,26,27], there is often a limited involvement of medical experts in their development [26,28,29], their information may not be regularly updated [25], and there is a general lack of oversight and regulation of what is available [26]. Further, the users of mHealth apps rarely verify the credibility of the developers, focusing instead on user ratings. User ratings show a limited correlation with an app having an evidence base [5]. Previous research has also indicated that there is a lack of transparency regarding organizational affiliations, with fewer than half of the publicly available cancer apps describing this [30,31]. Considering this, several reviews of mHealth cancer-related apps have been undertaken. These have mainly focused on apps designed for people with specific cancers (e.g., breast) [26,29,32,33,34] or the published literature, rather than the publicly available apps [3,11,35]. While a review conducted in 2020 evaluated symptom-tracking apps in oncology [36], to our knowledge, no studies have reviewed apps relevant across cancer populations that include any interactivity feature (not only symptom tracking). To address this gap, the aim of this study was to evaluate the quality and usefulness of free, publicly available apps for adults with cancer that include a tailored, interactive feature to support them in managing their illness.

## 2. Materials and Methods

Where relevant, the methods followed the Preferred Reporting Items for Systematic Reviews and Meta-Analyses (PRIMSA) guidelines to introduce rigor [37]. These include explicit inclusion/exclusion criteria and a description of the information sources and search strategy, as well as details about the selection process of the apps and data collection, extractions, and analysis.

### 2.1. App Sample

The apps of interest were those that patients would be likely to identify themselves. Therefore, the focus was on apps that were free of charge (as payment is a barrier to patients using an app) [25,38,39], did not require an institutional login (anyone could access the app or login easily obtained upon request), were designed specifically for adults with cancer, were available in either English or French, were updated within two years of the search, provided information that was generally applicable across clinical settings and cancer types, referenced coping with illness, included at least one interactive feature in which the user inputted information and the app tailored its content accordingly, and were available in both the Apple and Google play stores. As both Apple for IOS devices and Google play for Android devices are used by a large share of the market [40], the aim was to ensure the apps retained would be accessible to most users. Apps that were in-development or the beta stage, were unrelated to cancer (e.g., astrology), those targeting healthcare professionals or students, or those focused solely on cancer prevention, awareness, or detection were excluded.

### 2.2. Search Strategy

An initial search was conducted in June 2020 and then updated in June 2022 to identify the apps published in the interim two years. In 2020, the Google Play store was searched using a Samsung Galaxy S6, then the availability of the apps was checked in the Apple Store. The following search terms were used: cancer, cancer patient, cancer patient app, mHealth cancer, cancer aid, cancer support, apps for cancer, cancer management, and help with cancer. In 2022, the same search terms and methods were used. Prior to the searches, the cache and search history on the app stores were cleared to minimize the results tailored to the phone user.

After searching the apps stores, the search terms were entered into the Google search engine. In 2020, the first ten pages of results were verified; however, as few new apps were returned beyond the first two pages, the 2022 search was restricted to only these initial pages [41]. Google Chrome’s Incognito function was used to minimize bias. In 2020, the search for apps in the Google Play store was completed by one author (AA) and the Google Web search by another author (SW). The availability of the identified apps in the Apple Store was then also verified by one of these authors (SW). In 2022, the updated searches were all completed by one author who had been involved in the initial search (SW). All the app titles returned from the searches were entered into a Microsoft Excel spreadsheet.

### 2.3. Screening for Eligibility

After de-duplication, all the identified apps were screened in three stages: by title, then by description and preview screenshots, and finally through a download and verification of the app’s entire content. In the initial 2020 search, the screening at the title stage was completed by one author (AA) and the screening at the second and third stages were completed independently by two authors (AA and SW), who then met to compare their results. Few discrepancies were identified, and consensus was sought through discussion between the two authors. Any uncertainties were discussed at team meetings. If there was insufficient information available at one stage to assess the inclusion/exclusion criteria, the app was carried forward to the next stage. Prior to publication, the availability of the included apps was verified. Any previously retained apps that were no longer available were removed from the sample.

### 2.4. Data Collection

The included apps were then evaluated for their quality and usefulness. Each app identified in 2020 was evaluated independently by two authors (AA and SW). The scores were compared and any discrepancies were discussed until a consensus was reached between the raters. In 2022, one of the two authors (SW) involved in the initial extraction completed the updated data collection. Any questions were addressed at team meetings.

#### 2.4.1. Quality

Each app was evaluated for its quality using the standardized Mobile App Rating Scale (MARS) [42]. The MARS is a validated tool developed as a multi-dimensional measure for rating the quality of mobile health apps. The MARS includes 23 items across five sections: (a) engagement (five items), (b) functionality (four items), (c) aesthetics (three items), (d) information quality (seven items), and (e) subjective quality (four items). The first four sections were included in this evaluation, as their completion was validated for rating by the researchers (not end users), and only the last section should be completed by end users (excluded from this evaluation). All the items were scored from 1 = low to 5 = high according to the MARS guidelines. Although the MARS allows for some items to be scored “not applicable” (N/A), the team deemed all the items to be relevant to the apps included in this study. If an app did not address an item, it was scored a 1 to reflect its lower quality. Items 15, 16, and 17 in the information quality section were completed by a registered nurse. The section scores for each app, as well as the mean overall score, were calculated.

#### 2.4.2. Usefulness Checklist

The authors were unable to identify an existing usefulness measure for cancer apps. As such, a 16-item usefulness checklist was developed based on seven studies (including a systematic review) [25,26,43,44,45,46,47] describing the features of mHealth apps that adult cancer patients reported as desirable and useful. The 16 items of the usefulness checklist were grouped into four categories: communication features (four items, e.g., communication with healthcare professionals (HCP) and connecting with other patients), tracking (four items, e.g., tracking physical symptoms/side effects), cancer information (five items, e.g., general information about cancer and information on managing symptom/side effects), and practical app features (three items, e.g., privacy protocol). Each item was scored as 0 = not present or 1 = present, with a maximum score of 16.

#### 2.4.3. Characteristics Checklist

The app characteristics were also extracted. This information included: the date the app was last updated, country of origin, language(s) in which the app was available, target audience(s) (patients, caregivers, and/or healthcare professionals), learning styles (written, visual, and/or audio), type of publisher (healthcare institution or technology-based company), and app store star rating in both the Apple and Google Play stores. The app store star ratings were added to reflect the end-user reviews of the apps. In this case, if an app did not receive enough reviews (i.e., no rating available in app store), the apps were given a score of N/A. Additionally, the country in which an app was developed was not always indicated. In such cases, if the developers were named, a search was conducted for their location and included as a likely proxy. The characteristics of the apps were verified again in July 2023 to ensure the most updated information was available before publication. At this stage, two apps, Lyfe and Oncopadi, were removed from the sample, as they were no longer available in either app store.

### 2.5. Data Analysis

The descriptive analyses were undertaken using Microsoft Excel. The means and standard deviations of all the items, subsections, and totals of the MARS and usefulness checklists were calculated for each app, as well as across the sample. The apps were ranked based on these results and the findings are otherwise detailed descriptively.

## 3. Results

### 3.1. Search Results

**Figure 1:**
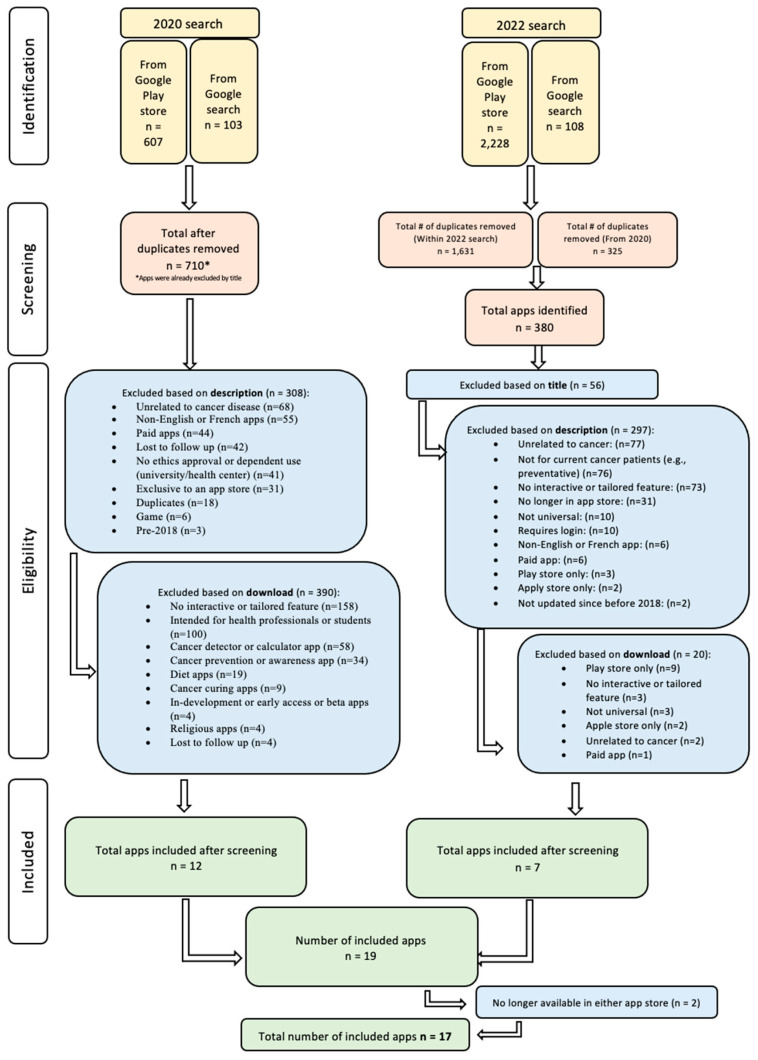
PRISMA Flow Diagram.

The results from the 2020 and 2022 searches are presented separately in Figure 1. Taken together, the searches returned 3046 apps. After duplicates were removed and the titles were screened (*n* = 2012), 1034 apps were screened by description. Of these, 605 were excluded based on description. The main reasons for exclusion were: requiring payment, unrelated to cancer, non-English or French, designed to prevent cancer, and no interactive or tailored features. A further 410 apps were excluded at the download stage. The main reasons for exclusion were: no interactive or tailored feature, intended for health professionals or students, or only available in one store. With 2 apps no longer available in either app store, a total of 17 apps were included from both the 2020 and 2022 searches. All apps were last accessed on 19 July 2023.

### 3.2. Description of App

See Table 1 for a description of the apps’ characteristics. Over half of the apps were updated by their respective developers in 2023 (*n* = 10), whereas most others were updated in 2022 (*n* = 5). The remaining two apps, Mindful USC and Virtual Hope, were updated in 2021 and 2019, respectively. Nearly all the apps were from the United States (*n* = 15), and the remaining two were from the United Kingdom (UK).

Ten apps were only available in English, whereas the other seven had options for more than one language, most often Spanish and French. Notably, Medisafe was available in 16 languages. In total, 4 apps were designed for use by patients living with cancer, whereas 12 targeted both patients and their caregivers, and 1 app was for patients, caregivers, and healthcare professionals (Cancer.net, accessed on 12 May 2023). The apps designed for patients and their caregivers (*n* = 12) fell into three categories: (a) five apps (Outcomes4me, Belong: Beating Cancer Together, Cancer.net, Pancreatic Cancer Action, and CancerAid) allowed caregivers to sign in to receive information on helping them with their caregiving role, with information targeted specifically towards the caregivers; (b) five apps (PatientMpower, HeadHelp: Self Care & Vent, Medisafe, LLS Health Manager, and The Cancer Coach) allowed caregivers to create an account to monitor what the patients inputted and used on the app (no information was offered to the caregiver themselves); and (c) two apps (Mindful USC and Smiling Mind) acknowledged in the description that caregivers could help patients with using the app (but no caregiver account and no information for the caregiver).

Additionally, the content was delivered to the users in a variety of ways, including written only (*n* = 3), written and graphics (*n* = 10), written, and graphics and audio (*n* = 4). Nine apps were developed by healthcare institutions, one by an educational institution, and the remaining seven were from private companies. In terms of the star ratings (out of 5) in both app stores, the average star rating for all the apps was 4.24/5. The top star rated apps were Smiling Mind (4.8), Belong—Beating Cancer Together (4.75), Bezzy BC (4.65), and Medisafe (4.65).

### 3.3. App Quality Ratings

The overall average MARS score for all 17 apps was 3.62/5 (SD 0.26, range: 3.14–4.06) (see Table 2, detailed scoring in Supplementary Materials). The top five highest scoring apps on the MARS were Outcomes4me (4.06), Medisafe (3.95), Virtual Hope Box (3.95), OWise—Prostate Cancer Support (3.94), and HeadHelp (3.94). Of note, although OWise—Prostate Cancer Support is designed for those with prostate cancer, its content is applicable to all patients with cancer, and was included.

When examining each section of the MARS separately, out of a possible score of five, the apps scored the highest on the functionality (3.99) and aesthetics (3.75) sections. Medisafe (4.75) and Cancer.net (4.50) were the most functional apps (e.g., easy to learn, use, and navigate with a logical flow and gestural design). Outocmes4me, LLS Health Manager, and HeadHelp: Self Care & Vent all scored 4.67, the highest score in terms of aesthetics (e.g., overall visual appeal).

The lowest-scoring sections on the MARS were information quality (3.26) and engagement (3.51). Within the information section, “evidence-based” (2.24) and “visual information” (2.65) were the items least addressed. To receive full points for being “evidence-based”, the apps would have to have been trialed, with their outcomes evaluated in three or more high-quality randomized controlled trials (RCTs) with a positive result. Three apps (Bezzy BC, Belong—Beating Cancer Together, and Cancer.net) received a score of 3/5 (one was included in a systematic app review (*n* = 1) and two in descriptive analyses). Two apps (Virtual Hope Box and Smiling Mind) received a score of 4/5 (these apps were evaluated in either one and two RCTs, respectively) and only two (PatientMPower and Medisafe) received a score of 5/5 (the first was evaluated in four RCTs and other acceptability and feasibility studies; the second in six RCTs and included in systematic reviews). The remaining ten apps in our sample had not been evaluated (score of 1/5). For information quality, Virtual Hope Box (4.14) scored the highest, followed by Medisafe (3.71) and OWise—Prostate Cancer Support (3.71). Of note, the “engagement” section scored low, even though all the apps had to have at least one interactive tailored feature. This could be attributed to the two other items in the engagement section scoring low (e.g., entertainment and interest). The most engaging app was Virtual Hope Box (4.40).

Most of the top-scoring apps on the MARS were updated in the past year, except for Virtual Hope Box. Only two of the top-scoring apps (Medisafe and Virtual Hope Box) were available in more than one language. Only Outcomes4me had tailored educational content for the user. Four of the top scoring apps had a self-monitoring feature and included tailored feedback, with only Virtual Hope Box lacking this element.

### 3.4. Usefulness Checklist

The average total usefulness score across the sample was 50% (SD 2.5, range: 31–88%) (see Table 3), indicating that most apps included only half the desired features identified by patients. With a score of 87.5%, Cancer.net was the highest-scoring app. On average, the apps included 27% of the desired communication features, 46% of the desired tracking features, 51% of the desired cancer information, and 86% of the desired practical app features.

The lowest-scoring items on the usefulness checklist were: communication with healthcare professionals (*n* = 1, The Cancer Coach), having a glossary of cancer terms (*n* = 2, OWise and Patient Communicator), and including a question prompt list (*n* = 4, OWise, LLS Health manager, Cancer.net, and Patient communicator).

The highest-scoring items on this checklist were: having a privacy protocol (*n* = 17), free to access to all parts of the app, as some other apps were free to download but required payment for additional features (*n* = 14), and being password protected/requiring a PIN (*n* = 13). The overall top five rated apps on this checklist were Cancer.net (88%), OWise (63%), Outcomes4me (63%), Belong—beating cancer together (63%), and CancerAid (63%). 

## 4. Discussion

Seventeen mHealth apps with interactive and tailored features were evaluated for patients with cancer. The apps were reviewed to identify those of the highest quality using a validated tool, as well as a usefulness checklist, to inform recommendations for patient use. The key findings were that (a) unexpectedly, most apps were designed to be used by patients and their caregivers; (b) the area of highest quality was “functionality”; (c) the higher-quality apps were not necessarily the most useful ones; and (d) most apps were not evidence-based or did not accommodate different learning styles.

Most apps were developed for use by both patients and their caregivers. This demonstrates an increased recognition of the important role that caregivers play in patients’ care and the management of cancer-related challenges. Caregivers provide 80% of the care for adults with chronic diseases [48], positively affecting patients’ health outcomes [49] and reducing demands on the healthcare system, with an estimated economic value of billions of dollars every year [50]. However, caregivers often do not receive the information and support they need [51], which may lead to a high burden [52]. mHealth resources offer benefits for caregivers, including a reduction in the caregiver burden and improved coping with the physical and mental health challenges related to the caregiver role [53]. It was surprising that so many apps were inclusive of caregivers when describing their target users, as the search was for patients specifically. This stands out particularly in comparison to another study that evaluated the quality (also using the MARS tool) of 24 completely different apps for caregivers of adults living with cancer [54]. The current study complements this to provide a more compressive list of apps that may be beneficial for supporting caregivers.

Another notable finding was that the highest-rated section on the MARS was “functionality”, which is consistent with other studies that have evaluated cancer mHealth apps [32,33,36]. Functionality refers to the ease with which users can learn and navigate through the app. This is likely associated with the rise in popularity of electronic health technology and the increased need for patients to navigate the resources and information available to them [55].

The average usefulness score of the top five highest-quality apps was 46%. Where two of the highest-quality apps were among the most useful (Outcomes4me and OWise), the other top three scored below average on the usefulness checklist. This indicates that, despite their level of quality, apps do not include many of the features desired by patients. The apps included communication features the least. Effective communication between patients, caregivers, and their healthcare professionals has been shown to reduce patient burden [56]. Potentially, the mismatch between the apps’ content and the patients’ desired features might explain the low adherence to apps over time [57]. The average MARS score among the top five most useful apps was 3.69/5. As noted, OWise and Outcomes4me were the overall highest-scoring apps, taking both quality and usefulness into consideration. Of these two apps, OWise did not have enough reviews to receive a star rating, and Outcomes4me had a star rating of 3.65, which was below the sample average. None of the highest-star-rated apps scored highly for usefulness.

Previous app evaluation studies have also found that the lowest-rated quality sections are “information” [33,36] and “engagement” [32]. The “information” section of the MARS focuses on the accuracy of the app description, goals, quality and quantity of the information, visual information, credibility, and evidence base. Within this section, the lowest-scoring item was “evidence-based”, flagging that many apps were not trialed/tested in published scientific literature. Potentially, the apps were not evidence-based because those reviewed in trials are not then made available publicly [3]. Most apps in this study were developed by health organizations that would not typically carry out trials to test the apps, but instead focus on their responsiveness to clinical and patient needs and their implementation. In addition to not being evidence-based, the lack of “visual information” was another reason for the low scores on the information section of the MARS. Many apps did not have visual explanations of concepts through charts/graphs/images or videos. This speaks to a lack of accommodation of different learning styles [58]. This finding emphasizes the need for more varied methods to promote uptake by users and to help retain information in mHealth apps.

The items in the “engagement” section of the MARS were entertainment, interest, customization, interactivity, and appropriate for the target audience. The lowest-scoring item was “entertainment”, indicating that many apps were not fun to use or would not stimulate repeated use and lacked features such as gamification. Though less common, apps with gamification to promote health behavior changes tend to be more effective than those without gamification [59,60]. Gamification is effective when it includes feedback on activity, self-monitoring, and the reinforcement of inputted information from search functions [61]. Although studies have shown that few health apps have been successful in improving patient outcomes [57,61], these findings contribute to enhancing our understanding of what would make future apps more effective in achieving this goal.

### Limitations

This study was conducted using reproducible and transparent methods to ensure rigor. Despite this, some limitations are noted. A patient evaluation of the apps was not within the scope of this paper. However, to palliate this limitation, we included a usefulness checklist based on end users’ perspectives in our evaluation, as well as star ratings. Due to the high turnover of apps, in the time between when the search was completed and the publication of this paper, more apps may have been developed. The apps in this study were all still available in both the Apple and Google play app stores in July 2023. Apps requiring an institutional login could not be evaluated. The authors contacted the developers of three known apps requiring an institutional log in, but access was not granted for this evaluation. Apps that required payment to access any (rather than only some) of their content were excluded, as payment is a barrier to access [25,38,39]. Potentially, apps requiring payment might be of a higher quality and/or usefulness. The apps’ readability was not evaluated, as there was not enough written content to complete this analysis. There is currently no validated suitability measure available for the particularities of app content. The development of such a measure is a potential area for future research. Last, since the team is bilingual, we were open to evaluating all apps in either English or French. However, all the search terms were in English.

## 5. Conclusions

This study evaluated 17 apps for patients with cancer, identifying the key strengths and weaknesses of each in terms of their quality, usefulness, and characteristics. Based on the findings, patients and healthcare professionals can identify the top-rated apps. While many mHealth apps still lack both high-quality information and patients’ desired features, those that scored the highest were: Outcomes4me and OWise (in terms of both quality and usefulness), as well as Cancer.net (in terms of usefulness). These findings may be used to inform future research for developing interactive mHealth apps that better address patient needs.

## Figures and Tables

**Table 1:** Characteristics checklist.

Characteristics	My Cancer Tracker (Health Stack, LLC)	OWise—Prostate Cancer Support (Px HealthCare Group Ltd.)	Bezzy BC (Healthline)	PatientMpower (PatientMpower)	HeadHelp: Self Care & Vent (Helponymous, LLC)	Outcomes4me	Medisafe	LLS Health Manager	Virtual Hope Box (VHB)	TheCancer Coach	Mindful USC	Smiling Mind	Belong: Beating Cancer Together	Cancer.net	Pancreatic Cancer Action (PCA)	CancerAid	Patient Communicator
**Last updated**	1 January 2022	1 January 2022	1 January 2023	1 January 2023	1 January 2023	1 January 2023	1 January 2023	1 November 2022	1 January 2019	1 May 2022	1 January 2021	1 January 2023	1 January 2023	1 January 2023	1 January 2023	1 January 2023	1 January 2023
**Country**	United States	UK	United States	United States	United States	United States	United States	United States	United States	United States	United States	UK	United States	United States	United States	United States	United States
**Language**	English	English	English	English, Danish, Dutch, French, German, Greek, Italian, and Spanish	English	English	English, Arabic, Danish, Dutch, Finnish, French, German, Hebrew, Italian, Japanese, Korean, Portuguese, Russian, Simplified Chinese, Spanish, and Turkish	English, French, and Spanish	English, German, Japanese, Polish, Simplified Chinese, and Spanish	English	English	English	English, French, German, Hebrew, and Spanish	English and Spanish	English, French, German, and Spanish	English	English
**Target audience**	Patients with cancer	Patients, applicable to all cancer	Patients, applicable to all cancer	Patients and caregivers	Patients and caregivers	Patients and caregivers	Patients and caregivers	Patients and caregivers	Patients and caregivers	Patients and caregivers	Patients and caregivers	Patients and caregivers	Patients and caregivers	Patients, caregivers, and healthcare providers	Patients and caregivers	Patients and caregivers	Patients
**Learning styles**	Visual, Written	Visual, Written	Written, Visual	Written	Visual, Audio, Written	Written, Visual	Written	Written, Visual	Written, Visual, Audio	Written, Visual	Written, Visual	Written, Visual, Audio	Written	Written, Visual	Written, Visual	Written, Visual	Written, Visual, Audio
**Publisher**	LLC (Health Stack	Sourced from a healthcare institution	Sourced from a healthcare institution	Sourced from a healthcare institution	Appcano LLC	Sourced from a healthcare institution	Medisafe	Sourced from a healthcare institution	National Centre for Telehealth & Technology	Coaches Anywhere LTD	Educational institution	Smiling Mind	Sourced from a healthcare institution	Sourced from a healthcare institution	Sourced from a healthcare institution	AON, Lendlease, MinterEllison	Sourced from a healthcare institution
**Reviews (# stars/5)—** **First row = Apple** **Second row = Google Play** **Third Row = Average**	N/A	N/A	4.7	N/A	4.6	3.3	4.7	N/A	3.8	N/A	4.8	4.7	4.8	4.6	N/A	4	N/A
	N/A, 10,000+ downloads	N/A, 500+ downloads	4.6, 10 K+	3.8, 5 k+ downloads	4, 10 k+ downloads	4, 100 K+	4.6, 5 M+	3.9, 1 k+	N/A, 1 k+	N/A, 50 k+	4.2, 1 k+	4.9, 1 M+	4.7, 100 k+ downloads	4.2, 10 k+	N/A, 500+	2.9, 5 k+	4.5, 5 k+
	N/A	N/A	4.65	3.8	4.3	3.65	4.65	3.9	3.8	N/A	4.5	4.8	4.75	4.4	N/A	3.45	4.5

**Table 2:** MARS scores by app.

	My Cancer Tracker	OWise	Bezzy	PatientMPower	HeadHelp	Pt Communicator	USC	LLS	Medisafe	Outcomes4me	PCA	VHB	Belong	Cancer Coach	Canceraid	Smiling Mind	Cancer.net	Average Score	Standard Deviation
**Section A: ** **Engagement**																			
**Mean**	3	3.8	3.8	2.8	4.2	2.6	3.8	3.6	3	4.2	4	4.4	4.2	3.2	3	3.4	2.6	**3.6**	**0.64**
**Section B: ** **Functionality**																			
**Mean**	4	4.3	4	3.8	3.5	3.5	4	3.8	4.8	4.5	3	4.3	4	4.3	4	3.8	4.5	**3.97**	**0.44**
**Section C: ** **Aesthetics**																			
**Mean**	3	4	3	3.7	4.7	3.3	3.3	4.7	4.3	4.7	3.7	3	3.3	3.7	3.7	4	3.7	**3.67**	**0.58**
**Section D: ** **Information**																			
**Mean**	2.6	3.7	3.3	3.4	3	3.6	3	3	3.7	2.9	3.1	4.1	2.7	3.6	2.7	3.6	3.4	**3.25**	**0.43**
**Total**	**3.1**	**3.9**	**3.5**	**3.4**	**3.8**	**3.3**	**3.5**	**3.8**	**3.9**	**4.1**	**3.5**	**3.9**	**3.6**	**3.7**	**3.3**	**3.7**	**3.5**	**3.6**	**0.27**

**Table 3:** Usefulness checklist ^1.^

Categories	Items	My Cancer Tracker (Health Stack, LLC)	OWise—Prostate Cancer Support (Px HealthCare Group Ltd.)	Bezzy BC (Healthline)	PatientMpower (PatientMpower)	HeadHelp: Self Care & Vent (Helponymous, LLC)	Outcomes4me	Medisafe	LLS Health Manager	Virtual Hope Box (VHB)	The Cancer Coach	Mindful USC	Smiling Mind	Belong: Beating Cancer Together	Cancer.net	Pancreatic Cancer Action (PCA)	CancerAid	Patient Communicator	Mean per Category (/17)	Mean per Category (%)
Communication features	#1: Communication with HCPs	0	0	0	0	0	0	0	0	0	1	0	0	0	0	0	0	0	1	5.88%
	#2: Reporting to HCPs	0	1	0	1	0	0	1	1	0	0	0	0	1	1	1	1	0	8	47.06%
	#3: Connect with other patients	1	0	1	0	1	1	0	0	0	0	0	0	1	0	0	0	0	5	29.41%
	#4: Question prompt list	0	1	0	0	0	0	0	1	0	0	0	0	0	1	0	0	1	4	23.53%
	AVERAGE in this section	25%	50%	25%	25%	25%	25%	25%	50%	0%	25%	0%	0%	50%	50%	25%	25%	25%	4.5	**26%**
Tracking	#5: Tracking physical symptoms/side effects	1	1	0	1	0	1	1	1	0	0	0	0	0	1	1	1	1	10	58.82%
	#6: Tracking psychosocial symptoms/side effects	1	1	0	0	1	1	0	0	0	0	1	1	0	1	0	1	0	8	47.06%
	#7: Tracking medications	1	0	0	1	0	1	1	1	0	0	0	0	0	1	1	0	0	7	41.18%
	#8: Tracking appointments	1	1	0	1	0	0	0	0	0	0	0	0	1	1	1	0	0	6	35.29%
	AVERAGE in this section	100%	75%	0%	75%	25%	75%	50%	50%	0%	0%	25%	25%	25%	100%	75%	50%	25%	7.75	**46%**
Cancer information	#9: General information about cancer	1	0	1	1	0	1	1	1	0	1	0	0	1	1	1	1	1	12	70.59%
	#10: Information on managing symptom/side effects	0	0	0	1	1	1	0	0	1	1	1	1	1	1	1	1	0	11	64.71%
	#11: Information on managing emotional health	0	0	0	0	1	1	0	0	1	1	1	1	1	1	0	1	0	9	52.94%
	#12: Glossary of cancer terms	0	1	0	0	0	0	0	0	0	0	0	0	0	0	0	0	1	2	11.76%
	#13: Linking to cancer support services	0	1	1	0	0	1	0	1	0	0	0	1	1	1	0	1	1	9	52.94%
	AVERAGE in this section	20%	40%	40%	40%	40%	80%	20%	40%	40%	60%	40%	60%	80%	80%	40%	80%	60%	8.6	**51%**
General practical app features	#14: Free to access all parts of the app	1	1	1	1	0	1	1	1	1	0	1	0	1	1	1	1	1	14	82.35%
	#15: Privacy protocol	1	1	1	1	1	1	1	1	1	1	1	1	1	1	1	1	1	17	100.00%
	#16: Security	1	1	1	1	1	0	0	1	1	1	0	1	1	1	1	1	0	13	76.47%
	AVERAGE in this section	100%	100%	100%	100%	67%	67%	67%	100%	100%	67%	67%	67%	100%	100%	100%	100%	67%	14.6666667	**86%**
TOTAL (out of 16)		9	10	6	9	6	10	6	9	5	5	5	6	10	14	9	10	7	Mean score of apps	8
Total (%)		56.25%	62.50%	37.50%	56.25%	37.50%	62.50%	37.50%	56.25%	31.25%	31.25%	31.25%	37.50%	62.50%	87.50%	56.25%	62.50%	43.75%		**50.00%**

^1^ Note. Developed based on studies [25,26,43,44,45,46,47].

## Data Availability

Available in Supplementary Materials.

## Supplementary Materials

The following supporting information can be downloaded at: https://www.mdpi.com/article/10.3390/curroncol30080518/s1, Table S1: Usefulness checklist; Table S2: Total MARS scores, Table S3: Characteristics checklist.
